# Dietary Patterns and Brain Health in Middle-Aged and Older Adults: A Narrative Review

**DOI:** 10.3390/nu17091436

**Published:** 2025-04-24

**Authors:** Jamie A. Seabrook, Abolfazl Avan, Colleen O’Connor, Harry Prapavessis, Lindsay Nagamatsu, Jasna Twynstra, Saverio Stranges, Arlene MacDougall, Vladimir Hachinski

**Affiliations:** 1 Department of Epidemiology & Biostatistics, Western University, London, ON N6G 2M1, Canada; aavan2@uwo.ca (A.A.); saverio.stranges@uwo.ca (S.S.); arlene.macdougall@sjhc.london.on.ca (A.M.); vladimir.hachinski@lhsc.on.ca (V.H.); 2 Department of Paediatrics, Western University, London, ON N6A 5W9, Canada; 3 Brescia School of Food and Nutritional Sciences, Western University, London, ON N6G 2V4, Canada; colleen.oconnor@uwo.ca (C.O.); jasna.twynstra@uwo.ca (J.T.); 4 Children’s Health Research Institute, London, ON N6C 2V5, Canada; 5 Lawson Research Institute, London, ON N6A 4V2, Canada; 6 London Health Sciences Centre Research Institute, London, ON N6A 5W9, Canada; 7 Robarts Research Institute, Western University, London, ON N6A 3K7, Canada; 8 School of Kinesiology, Western University, London, ON N6A 3K7, Canada; hprapave@uwo.ca (H.P.); lnagamat@uwo.ca (L.N.); 9 Department of Family Medicine, Western University, London, ON N6G 2M1, Canada; 10 Department of Medicine, Western University, London, ON N6A 5A5, Canada; 11 Department of Clinical Medicine and Surgery, University of Naples Federico II, 80131 Naples, Italy; 12 Department of Psychiatry, Western University, London, ON N6C 087, Canada; 13 Department of Clinical Neurological Sciences, Western University, London, ON N6A 3K7, Canada

**Keywords:** brain health, Mediterranean diet, cognitive function, neurodegenerative diseases, omega-3 fatty acids, antioxidants, dietary patterns, MIND diet, DASH diet, Western diet

## Abstract

Diet has a profound impact on brain health, particularly in middle-aged and older adults, who are at increased risk of cognitive decline and neurodegenerative diseases. Various dietary patterns, including the Mediterranean diet (MedDiet), Dietary Approaches to Stop Hypertension (DASH), and Mediterranean-DASH Intervention for Neurodegenerative Delay (MIND) diets, have been linked to improved cognitive function. While the relative effectiveness of these diets on brain health is generally supported by evidence, variability in study results suggests that further research is needed to fully understand their effects across diverse populations. The objective of this descriptive narrative review is to examine the role of dietary patterns in supporting brain health in aging populations and to propose practical dietary strategies for promoting cognitive well-being. A comprehensive review of the existing literature was conducted on PubMed in October 2024, with no restrictions on language, publication date (1966–2024), or geographic location. A total of 18 articles were included in this review, covering the years 2013–2023. Studies assessing the impact of the MedDiet, DASH, MIND, and Western diets on cognitive function in middle-aged and older adults were prioritized. The research findings were synthesized to identify common and unique recommendations across these dietary patterns. The MedDiet consistently showed beneficial effects on cognitive health, including improved memory, processing speed, and long-term protection against neurodegenerative conditions. The DASH and MIND diets demonstrated potential benefits, particularly for specific cognitive domains, but the results were more mixed and inconclusive. In contrast, adherence to a Western diet was associated with negative cognitive outcomes, including cognitive decline and smaller brain volumes. These findings underscore the importance of adopting healthy dietary patterns as a modifiable lifestyle factor to support cognitive aging and inform future public health strategies and clinical guidelines.

## 1. Introduction

Who we are, what we do, and how we think, and feel are all shaped by the brain, underscoring the principle that “there is no health without brain health” [1]. Our brain influences every aspect of our lives—physical and mental health, social well-being, productivity, and creativity [2,3,4,5,6]. While brain health is essential at every stage of life, the aging global population presents significant challenges in preserving cognitive, mental, and social well-being in older age [7]. Among the greatest threats to brain health are stroke, heart disease, and dementia—collectively known as the “triple threat” [1]. Stroke and dementia, in particular, are the leading causes of neurological disorders and their associated burdens [8]. They share common risk factors with each other and with ischemic heart disease [9], which is the most impactful chronic condition that is not spread through infection. A meta-analysis further highlights the interconnectedness of these conditions, showing that both prevalent stroke (n = 36 studies) and incident stroke (n = 12 studies) are strong independent risk factors for all-cause dementia, with pooled hazard ratios of 1.69 (95% confidence interval: 1.49–1.92) and 2.18 (95% confidence interval: 1.90–2.50), respectively [10]. These findings highlight the critical need to address and prevent stroke and dementia together, rather than treating them as isolated conditions.

These conditions—stroke, heart disease, and dementia—not only share overlapping risk factors such as hypertension and physical inactivity, but are also significantly influenced by lifestyle factors like diet [11]. As such, diet represents a modifiable target for reducing the burden of these conditions and promoting brain health across the lifespan [12].

An integral brain health implementation strategy promotes cerebral, mental, and social well-being across the lifespan by enhancing cognition, emotional health, and social connections, while preventing brain disorders through advocating the five pillars of ABCDS—Activity, Blood Pressure Control, Connecting, Diet, and Sleep—at individual and community levels. Implementing strategies that promote brain health involves practical and community- and individual-level efforts aimed at preserving and enhancing brain function through evidence-based lifestyle changes [11]. These integrated approaches underscore the importance of preventative strategies—particularly diet—in promoting brain health and reducing the burden of age-related cognitive decline [13]. This article focuses on one of these pillars—diet [14].

A growing body of evidence suggests that diets such as the Mediterranean (Med-Diet), DASH (Dietary Approaches to Stop Hypertension), and MIND (Mediterranean-DASH Intervention for Neurodegenerative Delay) are associated with better cognitive outcomes [15,16,17,18,19,20]. However, these findings are not entirely consistent, with some studies observing variations in cognitive outcomes across different populations, including those with diverse baseline cognitive statuses. This highlights the complexity of diet’s role in brain health [21,22,23]. Research in this area spans a wide range of dietary patterns and populations. While some studies have focused on the adverse effects of Western-style dietary patterns—particularly in relation to cognitive decline and changes in brain structure [21,23]—others emphasize the neuroprotective potential of diets rich in plant-based foods and healthy fats, such as the Mediterranean and DASH diets [14,15,19]. Despite the accumulating evidence, several gaps persist in the literature. For instance, there are few studies that directly compare the effects of multiple dietary patterns [14,16], and much of the research focuses on older adults, with limited data available for middle-aged populations who could benefit from early dietary interventions. Additionally, the risk of neurodegenerative diseases like Alzheimer’s and Parkinson’s increases significantly with age [24,25], further emphasizing the need for preventive strategies. Another challenge in the field is the inconsistency in how cognitive outcomes are measured; some studies focus narrowly on specific cognitive domains such as memory or executive function, while others report global cognitive scores [19,23], making it difficult to draw generalized conclusions.

This descriptive narrative review aims to synthesize the current knowledge on the impact of diet on brain health, with a particular emphasis on middle-aged and older adults. This focus is justified by several factors: middle-aged and older adults have unique nutritional needs due to reduced metabolism and changes in digestion [26,27]; dietary habits play a crucial role in cognitive decline [16,17]; addressing diet and brain health in older adults has profound public health implications given the aging global population [28,29]; and this demographic may be more open to adopting dietary changes that support health [30].

## 2. Materials and Methods

### 2.1. Search Strategy

The comprehensive literature search was conducted on PubMed on 17 October 2024, using the PICOS framework, with no restrictions on language, publication date (1966–2024), or geographic location. The framework components included the following:

- Population: Humans (adults, older adults);
- Intervention/Exposure: Diet;
- Comparator and Outcomes: Dementia/cognitive impairment, brain/mental/social health;
- Study Design: Observational and experimental studies, systematic reviews, and meta-analyses.

The diet-related search terms included the following: “Diet, Healthy”[Mesh], healthy diet*[tw], healthy eating[tw], healthy nutrition[tw], prudent diet*[tw], “Diet Therapy”[Mesh], diet therap*[tw], dietary modification*[tw], restrictive diet[tw], restriction diet therapy[tw], restrictive diet therapy[tw], dietary restriction*[tw], “Dietary Approaches to Stop Hypertension”[Mesh], hypertension diet*[tw], DASH diet*[tw], Mediterranean[tw], MIND[tw], and Nordic[tw]. The complete search string can be found in Appendix A.

The search strategy yielded 3134 citations related to diet and brain health. After an initial screening of titles and abstracts, studies were assessed for eligibility based on predefined inclusion and exclusion criteria. To be included in this descriptive narrative review, articles needed to focus on the association between diet and brain health, with an emphasis on overall dietary patterns. Studies were selected if they examined outcomes related to neurocognitive function, neurodevelopment, neuroprotection, neurogenesis, or brain structure. A wide range of article types were considered, including observational and experimental studies, systematic reviews, and meta-analyses. Studies focusing exclusively on animal models or cellular mechanisms without any human data were excluded. Additionally, papers that did not explicitly address cognitive or neurological outcomes in relation to dietary intake or patterns were omitted. The reference lists of the included studies were screened to identify any additional relevant literature. 

As this was a descriptive narrative review, the screening, selection, and data extraction processes were conducted by a single author (JAS). No formal risk-of-bias assessment or quality appraisal was conducted, consistent with the descriptive and exploratory nature of this review. The relevant articles were reviewed, and the data were extracted and synthesized to identify trends and common findings. Table 1 provides a summary of all 18 included articles, which were categorized as follows: 11 cohort studies, 3 randomized controlled trials, 2 systematic reviews and meta-analyses, and 2 cross-sectional studies. Wherever possible, conclusions were drawn based on higher-quality evidence (e.g., randomized controlled trials) to provide more reliable and robust insights into the association between diet and brain health [31].

### 2.2. Conceptual Framework

This descriptive narrative review was designed to synthesize the current evidence on dietary patterns and their impact on brain health, with a particular emphasis on middle-aged and older adults. The review was intentionally focused on human population-level studies, aiming to provide actionable insights for public health and policy. As such, the scope of this review concentrated specifically on the association between dietary patterns and cognitive outcomes, excluding discussions on emerging mechanisms or individual mediators that might influence these relationships.

While the gut–brain axis, gut microbiota, and genetic factors have been identified as important modulators of diet’s effects on brain health, these topics were outside the scope of this review. We made the decision to focus on established, broadly applicable dietary patterns (e.g., MedDiet, DASH, MIND) and their associations with cognitive outcomes, with the understanding that mechanistic discussions regarding the gut–brain axis, genetic predispositions, and other individual-level factors are better suited to specialized reviews or studies. Our goal was to keep the synthesis focused on dietary interventions that can be translated into practical recommendations for public health and individual-level dietary strategies. This approach aligns with the review’s intent to inform policy and provide practical guidance for promoting brain health across the lifespan.

## 3. Results

In this section, the results are presented in two main categories: first, key nutrients that support brain health, and second, dietary patterns associated with cognitive function.

### 3.1. The Role of Nutrition in Brain Function

Nutrition is fundamental for brain function, impacting a wide range of areas including mood [39,50], cognition [51,52], and vital processes such as neuroprotection and neurogenesis [53].

Omega-3 fatty acids, especially eicosapentaenoic acid (EPA) and docosahexaenoic acid (DHA), are essential for preserving the structure and function of neuronal membranes, and include foods such as fatty fish (e.g., salmon, sardines), walnuts, and chia seeds. They play a crucial role in synaptic plasticity, a key process for learning and memory [54]. Additionally, omega-3s enhance the production of brain-derived neurotrophic factor, a protein that supports neuronal growth and differentiation, while also helping to mitigate inflammation in the brain—a factor linked to neurodegenerative diseases. In a recent systematic review and meta-regression analysis of controlled trials, omega-3 supplementation significantly raised brain-derived neurotrophic factor levels compared to the control group (pooled weighted mean difference of 1.01 μmol/L; 95% confidence interval 0.35 to 1.67; *p* = 0.003), and this increase was more pronounced for interventions > 10 weeks and doses ≤ 1500 mg/day [46].

Antioxidants—including vitamins C and E, flavonoids, and polyphenols—play a vital role in safeguarding the brain from oxidative stress, which can harm cells and contribute to cognitive decline [55]. Foods rich in antioxidants include berries, spinach, and dark chocolate. By neutralizing free radicals, antioxidants help to reduce cellular damage and inflammation [56]. Certain dietary antioxidants, such as polyphenols from blueberries and polyunsaturated fatty acids, can further enhance neurogenesis and improve cognitive function by promoting mitochondrial health [57].

B vitamins, particularly B6, B12, and folate, are crucial for energy production, neurotransmitter synthesis, and maintaining balanced homocysteine levels—elevations of which are associated with cognitive decline [58]. Foods high in B vitamins include whole grains (e.g., brown rice, quinoa), eggs, and leafy greens (e.g., spinach, kale). These vitamins facilitate the production of important neurotransmitters such as serotonin, dopamine, and norepinephrine, significantly influencing mood and cognitive functions [59]. Additionally, B vitamins are integral to the methylation process, which is essential for regulating gene expression and ensuring optimal neuronal function [58]. 

Vitamin D is increasingly recognized for its significant role in neurodevelopment and overall brain function [60,61]. Foods that are high in Vitamin D include egg yolks, fatty fish, and fortified foods (e.g., fortified milk and cereals). Research has shown that a deficiency in this vital nutrient is linked to a range of serious neurological conditions, including Parkinson’s disease and Alzheimer’s disease. Furthermore, low levels of vitamin D have also been associated with increased risks of depression and cognitive decline in adults [61]. 

Magnesium is a vital mineral involved in over 300 biochemical reactions in the body, playing a crucial role in processes that support brain health and overall well-being [62,63]. Magnesium-rich foods include leafy greens, nuts and seeds (e.g., almonds, pumpkin seeds), and legumes (e.g., black beans, chickpeas). With two-thirds of the population in the Western world failing to meet the recommended daily allowance of magnesium, this deficiency has been linked to migraine headaches, Alzheimer’s disease, stroke, hypertension, cardiovascular disease, and type 2 diabetes [63].

Zinc plays a vital role in synaptic plasticity and overall brain health [64,65,66]. Rich food sources of zinc include oysters, pumpkin seeds, and beef. Changes in brain zinc levels have been linked to several neurological conditions, including Alzheimer’s disease, depression, Parkinson’s disease, and Huntington’s disease [64,66]. Additionally, zinc is crucial for modulating various neurotransmitter systems and is essential for the proper functioning of brain-derived neurotrophic factor [67].

Polyphenols, abundant in fruits, vegetables, tea, and wine, possess powerful anti-inflammatory and antioxidant properties [68,69]. These compounds may enhance brain function by improving blood flow, reducing inflammation, and promoting neurogenesis [70]. Additionally, polyphenols have demonstrated a protective role against neurodegenerative diseases such as Parkinson’s and Alzheimer’s [71].

In summary, diets rich in these essential nutrients can support brain health through multiple mechanisms, such as reducing inflammation, promoting neurogenesis, and enhancing neurotransmitter function.

### 3.2. Dietary Patterns and Brain Health

In the following subsections, an overview and synthesis of dietary patterns commonly considered beneficial for brain health—MedDiet, DASH, and the MIND diet—alongside detrimental patterns like the Western diet, will be presented. An overview of these dietary patterns, as well as common and unique recommendations across brain-healthy diets, can also be found in Table 2.

#### 3.2.1. Mediterranean Diet

The MedDiet largely consists of fruits, vegetables, whole grains, legumes, nuts, seeds, herbs, and olive oil, whereas dairy products, fish, poultry, and wine are consumed in moderation, and red meat in low quantities [72]. In a recent systematic review investigating the association between dietary patterns in healthy middle-aged adults and concurrent or later neurocognitive function and brain health, two studies were reported from randomized controlled trials, eleven from longitudinal studies, and nine studies from cross-sectional research [12]. Both randomized controlled trials assessed the effects of adherence to the MedDiet on cognitive performance using cross-over designs and found improved (faster) processing speed after 8 weeks of intervention when compared to a low-fat control diet [47,48]. Additionally, longitudinal studies found that the MedDiet during midlife was beneficial for neurocognitive outcomes later in life. For example, higher adherence to the MedDiet was associated with better cognitive function and lower risk of poor cognition about 13 years later in 8009 older UK adults [44]. A one-point increase in adherence was equivalent to 1.7 fewer years of cognitive aging, with significantly lower odds of poor memory and processing speed (odds ratios 0.74–0.84; all *p* < 0.05). In a study of 16,948 Chinese adults [20], individuals in the highest quartile of an alternative MedDiet quality score had a 33% lower likelihood of developing cognitive impairment over a 20-year period, compared to those in the lowest quartile (odds ratio: 0.67; 95% confidence interval [0.59, 0.77]). In a cohort of 913 middle-aged and older Puerto Rican adults, adherence to a MedDiet was associated with higher 2-year cognitive function among those with type 2 diabetes [40]. Berti et al. [35] estimated that higher adherence to a MedDiet may provide up to 3.5 years of protection against brain aging and Alzheimer’s disease. In this 3-year imaging study of 70 cognitively normal adults aged 30–60 years, lower adherence was associated with greater declines in glucose metabolism and increased β-amyloid accumulation (*p* < 0.001), while no differences were observed in magnetic resonance imaging (MRI). Bhushan et al. [36] found that long-term adherence to a MedDiet pattern was strongly correlated with better subjective cognitive function in male health professionals and that it was the consumption of vegetables, fruits, nuts, and fish that had the greatest positive impact on cognitive function. Lastly, in a cohort of 10,670 women, greater adherence to an alternate MedDiet in midlife (median age = 59 years) was related to 46% greater odds of healthy aging (i.e., physical, mental, and cognitive health) among those surviving to older ages [43].

#### 3.2.2. DASH Diet

The DASH diet mostly consists of fruits, vegetables, low-fat dairy products, whole grains, legumes, nuts, and seeds, with poultry, fish, and lean meat consumed in moderation, and fats and oils in limited quantity [72]. As reported in the systematic review by Gauci et al. [12], there have been three longitudinal studies assessing adherence to the DASH diet during midlife and its impact on cognitive function. In the Boston Puerto Rican Health Study (n = 913; 42.6% with type 2 diabetes at 2 years), Mattei et al. [40] found that higher adherence to the DASH diet was associated with better 2-year memory function, word list learning, and Stroop test performance among participants without type 2 diabetes, after adjusting for baseline measures. However, DASH adherence was not linked to global cognitive function or executive functioning, regardless of diabetes status, highlighting the diet’s potential benefits for specific cognitive domains in non-diabetic individuals. In a prospective cohort study of high-socioeconomic-status Spanish adults aged over 55 years (n = 806), Muñoz García et al. [41] observed that adherence to the DASH diet was not significantly linked to cognitive improvements over six years, and that although some positive trends in cognitive changes were noted, the findings were predominantly consistent with null effects. In the Singapore Chinese Health Study, which followed 16,948 men and women aged 45–74 years at baseline (1993–1998) and reassessed them approximately 20 years later during the third follow-up visit (2014–2016), participants in the highest quartile of DASH diet adherence were 29% less likely to experience cognitive impairment compared to those in the lowest quartile (odds ratio: 0.71; 95% confidence interval: 0.62, 0.81) [20].

#### 3.2.3. MIND Diet

The MIND diet is made up of green leafy and other vegetables, berries, nuts, beans, whole-grain cereals, and olive oil, with fish, poultry, and wine consumed in moderation, and red meat, cheese, butter, margarine, fried foods, and fast foods only eaten in low quantities [72]. There have been three cohort studies assessing the correlation between the MIND diet and neurocognitive function. In a sample of 6011 men and women from France who were aged ≥ 60 years at baseline and without subjective memory complaints, greater adherence to the MIND diet was associated with lower subjective memory complaints among those aged 70+ years over a mean follow-up of 6 years [32]. This inverse relationship was stronger among those who were free of depressive symptoms (hazard ratio for highest vs. lowest adherence = 0.62; 95% confidence interval: 0.41–0.93). Another cohort study from Australia evaluated the association between the MIND diet and incidence of mild cognitive impairment [38]. Higher adherence to the MIND diet was linked to a 19% reduction in the odds of developing clinically diagnosed mild cognitive impairment over a 12-year follow-up period and the highest tertile of adherence experienced a 53% reduction in risk. Lastly, Muñoz García et al. [41] found that only high adherence to the MIND diet was beneficial for cognitive function 6 years later, and that the results were inconclusive for the MedDiet and DASH. Specifically, a 1.5-point increase in MIND score was associated with a 0.27-point improvement in cognitive function (95% confidence interval: 0.05–0.48), while changes for other diets were not statistically significant. Most recently, in a two-site randomized controlled trial of older adults (≥65 years) without cognitive impairment, but with a family history of dementia, cognitive function and brain imaging outcomes at 3 years did not differ significantly between participants following the MIND diet (n = 301) compared to those following a control diet (n = 303) with mild caloric restriction [34].

#### 3.2.4. Western Diet

The Western diet is a contemporary eating pattern typically marked by the frequent consumption of ultra-processed packaged foods, refined grains, red and processed meats, sugary beverages, candy, desserts, fried foods, conventionally produced animal products, high-fat dairy, and items containing high levels of fructose [73]. A 4-year longitudinal study of 255 individuals aged 60–64 years at baseline, conducted in Southeastern Australia [21], found that greater adherence to a Western dietary pattern was independently associated with smaller left hippocampal volumes, which refers to a reduction in the size of the hippocampus—a brain region important for memory and learning. This association persisted even after adjusting for age, gender, education, employment status, depressive symptoms, medication use, physical activity, smoking, hypertension, and diabetes [21]. In a cohort study of 13,588 adults from the U.S. without dementia at baseline (mean age = 54.6 years), the Western dietary pattern was not associated with cognitive decline 20 years later [37]. No significant differences were found in global cognitive change (z-score difference for highest vs. lowest adherence: −0.01; 95% confidence interval: −0.05 to 0.04) or dementia risk (hazard ratio = 1.06; 95% CI: 0.92–1.22). A cohort study involving 18,080 geographically diverse Black and White Americans aged 45 years and older revealed notable associations between dietary patterns and cognitive function. Diets rich in fried foods and processed meats were linked to lower cognitive scores 6–8 years later. In contrast, dietary patterns emphasizing plant-based foods, such as vegetables, fruits, and legumes, as well as those incorporating alcohol and salads (i.e., mostly green leafy vegetables, tomatoes, salad dressing, wine, and liquor) were associated with better cognitive performance [42]. A 10-year longitudinal study of 4847 individuals aged 55 years and older in China examined the relationship between dietary patterns and cognitive function. A protein-rich diet, characterized by the high consumption of milk, eggs, and soymilk, was positively associated with higher global cognitive and verbal memory scores. Conversely, a starch-rich diet, dominated by salted vegetables and legumes, was significantly linked to lower scores in these cognitive domains. Additionally, traditional Chinese dietary patterns, which include staples such as rice, pork, and fish, were positively associated with global memory scores, but showed no significant correlation with verbal memory [49]. Another prospective cohort study from China involving 4852 individuals aged 55 years and older found that a plant-based, iron-related dietary pattern—characterized by high consumption of fresh vegetables, wheat, legumes, beverages, offal, rice, and whole grains—was associated with poorer cognitive function among those with little to no meat intake (odds ratio for highest vs. lowest quartile = 1.50; 95% confidence interval: 1.17–1.93). This relationship was partially mediated by intake levels of iron, lead, and carbohydrates [45]. Finally, Akbaraly et al. [33] reported no significant association between a Western dietary pattern and the risk of developing dementia over a median follow-up of 24.8 years or with cognitive decline over an 18-year period.

## 4. Discussion

This review underscores the significant role that diet plays in brain health, with the MedDiet emerging as the most consistently supported dietary pattern in relation to improved cognitive outcomes. Research has linked higher adherence to the MedDiet with enhanced memory, processing speed, and long-term protection against cognitive decline, including a reduced risk of neurodegenerative diseases such as Alzheimer’s and dementia [35,44]. These benefits are often attributed to the MedDiet’s rich content of omega-3 fatty acids, antioxidants, and polyphenols, which support neurogenesis, reduce inflammation, and improve neuronal function [12]. However, despite this promising evidence, important methodological limitations temper the strength of these conclusions. In particular, most studies do not assess dietary adherence at follow-up [12]. This absence of longitudinal dietary tracking restricts our ability to determine whether the observed cognitive benefits are sustained over time or are influenced by potential fluctuations in adherence. Without regular assessments of dietary patterns throughout the study period, the long-term effects of the MedDiet remain uncertain. Future research would benefit from integrating repeated measures of dietary adherence to better understand the durability of these associations. Nonetheless, the overall body of evidence—particularly from observational studies, and a smaller number of randomized controlled trials—continues to suggest the beneficial role of the MedDiet in promoting brain health [12,72].

In contrast, evidence surrounding the DASH and MIND diets is less conclusive. The DASH diet shows some positive effects on cognitive performance, particularly in memory and learning, especially for individuals without diabetes [40]. However, the findings are mixed, with some research failing to show significant long-term cognitive benefits [41]. The inconsistent findings highlight the need for more rigorous study designs with longitudinal tracking of dietary adherence. Similarly, the MIND diet, which combines elements of the MedDiet and DASH, has demonstrated some potential in reducing cognitive decline and the risk of developing mild cognitive impairment [38]. However, this evidence is constrained by similar methodological shortcomings. As highlighted in the systematic review by Gauci et al. [12], studies such as those by Hosking et al. [38] and Muñoz García et al. [41] did not measure MIND diet adherence across the follow-up period. This lack of temporal dietary data limits our understanding of the stability of dietary patterns and their relationship with cognitive health over time. Therefore, while both the DASH and MIND diets show promise, particularly in specific populations or cognitive domains, the broader evidence base remains limited by study designs that fail to account for the dynamic nature of dietary behaviors. Future research should prioritize repeated measures of dietary adherence to better assess the durability and scope of cognitive benefits.

Conversely, adherence to a Western diet appears to have detrimental effects on cognitive health, including cognitive decline and smaller brain volumes [21]. The diet’s high inflammatory potential and lack of essential nutrients, such as omega-3s and antioxidants, may contribute to this negative impact [19]. Furthermore, the Western diet is associated with greater risks of cardiovascular diseases, which are in turn closely linked to cognitive decline and dementia [4]. The evidence strongly suggests that avoiding or minimizing Western dietary patterns may be an important strategy for preserving brain health as individuals age.

Overall, these findings indicate that the most impactful, cost-effective, and practical dietary strategies for improving brain health include increasing omega-3 intake through budget-friendly sources such as canned fish and seeds, boosting consumption of fruits, vegetables, and whole grains, and reducing processed foods and refined grains (see Panel Discussion Box). In addition to established diets, there is growing interest in the potential benefits of strategies that focus on replacing unhealthy diet components with healthier alternatives. For example, swapping ultra-processed foods high in sugars, unhealthy fats, and refined carbohydrates with nutrient-dense options like fruits, vegetables, whole grains, and lean proteins can improve overall brain health. This approach may help to counteract the negative impacts of poor diet quality while providing more sustainable long-term changes. Given the overwhelming evidence linking unhealthy diets with cognitive decline, focusing on such dietary swaps could offer a practical and effective strategy to support brain health in individuals at risk of neurodegenerative conditions.

**Figure 1:**
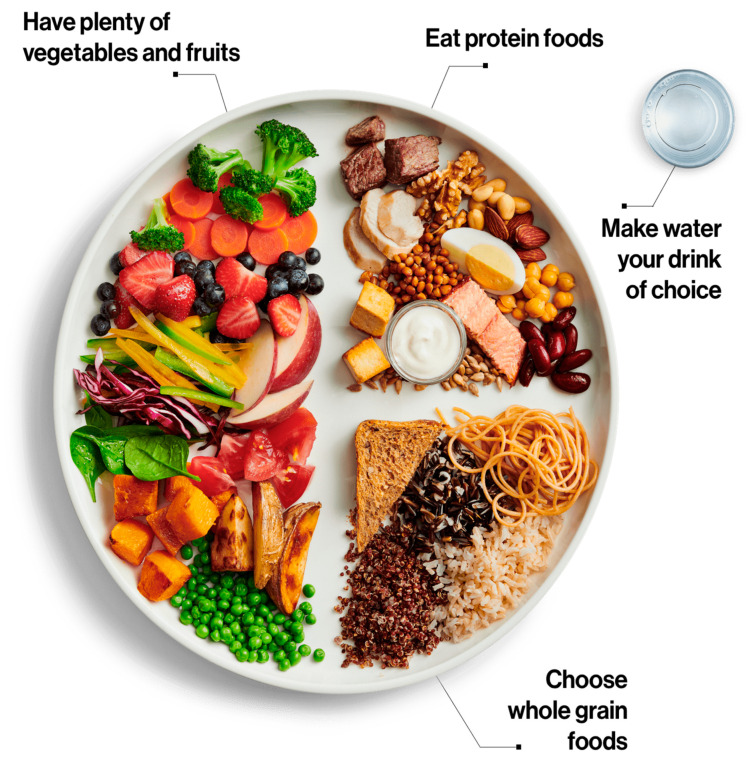
Eat a variety of healthy foods each day. Source: [74], modified, 24 March 2025. Adapted and reproduced with permission from the Minister of Health, 2025. Available at: https://food-guide.canada.ca/en/food-guide-snapshot/, accessed on 24 March 2025.

Another way to approach dietary intake and brain health is by considering how to divide the plate at each meal. For example, according to Canada’s food guide [74], one should aim to fill half of their plate with fruits and vegetables, a quarter with protein foods, and the remaining quarter with whole grains. Water is the ideal beverage, and incorporating more plant-based protein foods can also help support better health. See Figure 1.

While this narrative review provides a comprehensive synthesis of the relationship between diet and brain health, many studies relied on self-reported dietary patterns, which can be prone to misclassification. Additionally, the variation in study designs, durations, and populations included in the studies limits the generalizability of the findings. Future studies should aim to incorporate repeated dietary assessments, long-term follow-ups, and diverse populations to better understand the role of diet in cognitive aging. Furthermore, mechanistic studies exploring how specific nutrients within these dietary patterns influence brain health at the molecular and cellular levels would provide deeper insights into the underlying mechanisms of these effects.

In conclusion, this descriptive narrative review synthesized the current evidence on dietary patterns and brain health in middle-aged and older adults. While the MedDiet appears to have the most consistently reported benefits, the evidence supporting the DASH and MIND diets is less conclusive. These findings highlight the need for more comprehensive, long-term studies. Meanwhile, adherence to the Western diet appears to have a detrimental effect on cognitive function, underscoring the importance of adopting healthier dietary patterns for brain health. As research in this area progresses, it is essential to focus on refining study designs, ensuring regular dietary assessments, and investigating the molecular pathways that link nutrition to cognitive function.

Lastly, while this review focused on the “D” for diet within the ABCDS brain health framework, the interconnected nature of these pillars warrants further consideration. Diet can directly and indirectly influence other components—nutritional choices impact sleep quality, blood pressure regulation, and energy levels that support physical activity. For instance, diets rich in whole foods and low in added sugars may contribute to more restorative sleep and better mood regulation, which in turn enhance social engagement (“Connecting”) and motivation for exercise (“Activity”). Similarly, adherence to diets like the MedDiet or DASH is known to support healthy blood pressure levels. By considering diet not as an isolated factor, but as part of a synergistic system that supports brain health, interventions can be more holistic and impactful. Future research should explore these interconnections to better design multi-pronged strategies that align with the ABCDS framework.

## 5. Implications for Practice and Future Directions

The growing body of evidence linking diet and brain health highlights the urgency of translating findings into actionable, equitable strategies. As the global population ages, there is a pressing need to design dietary interventions that are both culturally relevant and scalable. For example, adapting dietary recommendations to local food preferences, reducing cost barriers, and leveraging technology—such as artificial intelligence and digital nutrition platforms—can help personalize guidance and support behavior change. The ABCDS framework provides a useful lens for this integration, especially when dietary strategies are aligned with improvements in physical activity, blood pressure management, connecting with others, and sleep.

Additionally, community-level efforts must be inclusive and supported by appropriate policy and infrastructure. Interventions that engage local organizations, healthcare providers, and caregivers are essential for sustainable implementation. Future research should also prioritize diverse populations and low-resource settings to address health disparities and ensure the broader applicability of findings. Finally, mechanistic studies investigating how specific nutrients and dietary patterns influence brain function at the cellular and molecular levels will be critical for refining and targeting future recommendations.

**Figure 2:**
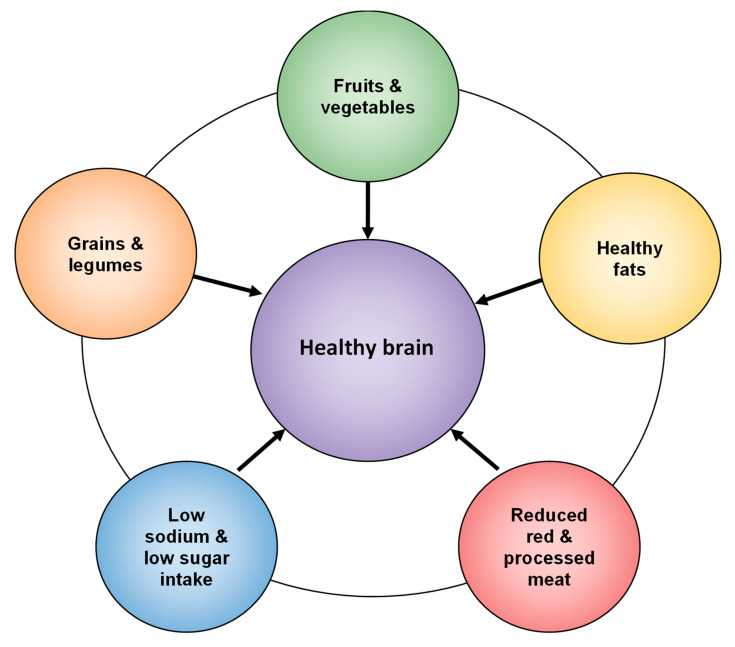
Brain-healthy dietary recommendations.

Figure 2 illustrates a conceptual model linking the brain to healthy dietary recommendations, while the Panel Discussion Box outlines practical and cost-effective dietary strategies, addresses cultural and socioeconomic factors, and explores the role of emerging technologies, policy integration, and equity-driven approaches. Together, these tools offer practical and scalable entry points for clinicians, policymakers, and individuals seeking to promote brain health through nutrition.

## Figures and Tables

**Table 1:** Overview of studies on dietary patterns and brain health in middle-aged and older adults (N = 18).

Author(s), Year	Objective	Study Design	Sample Size	Key Findings
Adjibade et al., 2019 [32]	To examine whether adherence to the MIND diet is associated with subjective memory complaints (SMC) in older adults.	Prospective Cohort.	6011 participants aged ≥ 60 years without SMC at baseline.	No significant association in full sample or ages 60–69. In adults ≥ 70 years, higher MIND adherence was associated with lower risk of SMC, especially after excluding those with depressive symptoms.
Akbaraly et al., 2019 [33]	To examine whether midlife diet quality is associated with later risk of dementia.	Population-based prospective cohort study with up to 25 years of follow-up.	8225 participants without dementia at baseline (mean age ~50).	No significant association between diet quality in midlife and incident dementia over long-term follow-up.
Barnes et al., 2023 [34]	To test whether the MIND diet with mild caloric restriction improves cognition compared to a control diet in older adults at risk of dementia.	Two-site, randomized controlled trial over 3 years.	604 participants (301 MIND diet, 303 control).	No significant difference in cognitive outcomes or brain MRI measures between MIND and control diet groups after 3 years. Both groups showed modest cognitive improvements.
Berti et al., 2018 [35]	To examine the effects of higher vs. lower adherence to a Mediterranean-style diet (MeDi) on Alzheimer’s disease (AD) biomarkers over 3 years.	Longitudinal study with clinical, neuropsychological, and imaging assessments at least 2 years apart.	70 cognitively normal adults (34 MeDi+ and 36 MeDi-), aged 30–60.	MeDi- group had lower glucose metabolism (FDG-PET) and higher β-amyloid load (PiB-PET) at baseline. Longitudinally, MeDi- showed greater declines in CMRglc and increases in PiB. No MRI effects. Higher MeDi adherence was associated with 1.5 to 3.5 years of AD protection.
Bhushan et al., 2018 [36]	To assess the association between long-term adherence to a Mediterranean diet and self-reported subjective cognitive function (SCF).	Prospective observational study.	27,842 men from the Health Professionals’ Follow-up Study, aged 40–75 at enrollment.	Men in the highest Mediterranean diet quintile had 36% lower odds of poor SCF and 24% lower odds of moderate SCF compared to those in the lowest quintile. Associations were consistent over time, with both past and recent diet contributing.
Dearborn-Tomazos et al., 2019 [37]	To examine the association of dietary patterns in midlife with cognitive function and dementia risk in later life.	Observational cohort study using data from the ARIC study (1987–2017).	13,588 participants (55.8% women), mean age 54.6 at baseline.	No association between midlife dietary patterns (Western or prudent) and cognitive function change over 20 years or risk of incident dementia. Participants with a Western diet had lower cognitive scores at baseline, but no significant long-term differences in cognitive change or dementia risk.
Hosking et al., 2019 [38]	To evaluate the relationship between the MIND and Mediterranean diets and the 12-year incidence of Alzheimer’s disease, vascular dementia, and mild cognitive impairment in Australia.	Longitudinal cohort study (PATH Through Life).	1220 participants (Canberra, Australia).	In adjusted models, the MIND diet was associated with reduced odds of cognitive impairment (OR = 0.47, 95% CI 0.24, 0.91) over 12 years, but the Mediterranean diet was not. The MIND diet’s protective effects appear geographically generalizable, though further studies are needed.
Lassale et al., 2019 [39]	To synthesize evidence on the link between diet quality and depression outcomes to guide future psychiatric healthcare.	Systematic review and meta-analysis of longitudinal and cross-sectional studies.	41 studies (20 longitudinal, 21 cross-sectional).	The Mediterranean diet showed the strongest association with a lower incidence of depression (relative risk 0.67). A lower Dietary Inflammatory Index was also linked to reduced depression incidence (relative risk 0.76). Other dietary indices (e.g., HEI, AHEI) showed similar trends. Adherence to a healthy diet, particularly the Mediterranean diet, appears to offer protection against depression.
Mattei et al., 2019 [40]	To examine associations between Mediterranean diet score (MeDS) and 2-year change in cognitive function based on type 2 diabetes and glycemic control status.	Longitudinal study (Boston Puerto Rican Health Study).	913 (42.6% with type 2 diabetes at baseline).	Higher MeDS was associated with greater improvement in global cognitive function in adults with type 2 diabetes (*p* = 0.016). This effect was significant for those with controlled glycemic levels and stable/improved control over 2 years, but not for those with uncontrolled or declined glycemic control. Healthy diets supported memory function in adults without type 2 diabetes.
Muñoz García et al., 2020 [41]	To study and compare associations of 5 dietary patterns (MDP, DASH, MIND, AHEI-2010, and PVD) with cognitive function.	Cohort Study.	806 participants.	The MIND diet and AHEI-2010 were beneficially associated with cognitive function. MDP, DASH, and PVD showed positive trends, but results were not statistically significant. The MIND diet appeared to modify cognitive function changes over time.
Pearson et al., 2016 [42]	To evaluate associations between dietary patterns and cognitive function in older adults.	Cohort study using data from the REasons for Geographic And Racial Differences in Stroke (REGARDS) cohort.	18,080 participants aged 45 and older.	Five dietary patterns were identified: convenience, plant-based, sweets/fats, Southern, and alcohol/salads. Greater consumption of the alcohol/salads pattern was associated with lower odds of cognitive impairment and higher cognitive function scores. The plant-based pattern was linked to better learning and memory scores. The Southern pattern, characterized by fried food and processed meats, was associated with lower cognitive scores.
Samieri et al., 2013 [43]	To examine the association between dietary patterns in midlife and the prevalence of healthy aging.	Cross-sectional observational study.	10,670 women.	Greater adherence to the Alternative Healthy Eating Index-2010 (AHEI-2010) and Alternate Mediterranean diet in midlife was associated with significantly higher odds of healthy aging (34% and 46%, respectively). This was particularly linked to better physical function and mental health. The study suggests that better diet quality in midlife is strongly linked to health and well-being in older age.
Shannon et al., 2019 [44]	To examine associations between MedDiet adherence and cognitive function in older UK adults. To investigate whether associations differ between individuals with high and low cardiovascular disease (CVD) risk.	Cross-sectional observational study.	8009 participants.	Higher adherence to MedDiet associated with better cognitive function. MedDiet adherence linked to lower risk of poor cognitive performance. A 1-point increase in Pyramid MedDiet score = 1.7 fewer years of cognitive aging. Stronger associations found in individuals at higher CVD risk.
Shi et al., 2019 [45]	To examine the association between iron-related dietary pattern (IDP) and cognitive function in Chinese adults.	Longitudinal study using data from the China Health and Nutrition Survey (CHNS) (1991–2006).	N = 4852, ≥55 years old.	High IDP intake was associated with poor cognition. The odds ratio for poor cognitive function across IDP quartiles was 1.00, 1.06, 1.24, and 1.50. The association was significant only among those with low or no meat intake. IDP was also positively associated with lead intake, mediating the relationship.
Sohouli et al., 2023 [46]	To investigate the effects of omega-3 supplementation on BDNF levels.	Random-effects meta-analysis of controlled trials.	Data from multiple trials.	Omega-3 supplementation significantly increased BDNF levels (pooled WMD 1.01 μmol/L, 95% CI 0.35–1.67, *p* = 0.003). The increase was more pronounced for interventions > 10 weeks and doses ≤ 1500 mg/day, especially in individuals under 50 years.
Wade et al., 2019 [47]	To assess the cognitive effects of a Mediterranean diet, including fresh, lean pork, in older adults.	24-week parallel cross-over trial comparing MedPork vs. low-fat control diet.	N = 35, aged 45–80, at cardiovascular risk.	MedPork led to improved processing speed (*p* = 0.01) and emotional role functioning (*p* = 0.03) compared to control. Diet was well tolerated.
Wade et al., 2020 [48]	To examine the cognitive and psychological effects of a Mediterranean diet with adequate calcium in an aging population.	Randomized controlled cross-over trial comparing MedDairy and low-fat control diet.	N = 41, aged ≥ 45, at cardiovascular risk.	MedDairy improved processing speed (*p* = 0.04) and several mood measures (Total Mood Disturbance, Tension, Depression, Anger, Confusion). No significant effects on memory, planning, or dementia risk scores.
Xu et al., 2018 [49]	To examine the association between dietary patterns, hypertension, and cognitive function in older adults.	Longitudinal study of CHNS data (1997–2006).	N = 4847 (10,658 observations), aged ≥ 55 years.	A protein-rich diet was linked to better cognitive scores; a starch-rich diet was linked to poorer scores. Hypertension was independently associated with lower cognitive function. Diet and hypertension both play roles in cognitive aging.

**Table 2:** Practical dietary recommendations for implementations based on common dietary patterns.

Overview of Dietary Patterns Mediterranean Diet (MedDiet) ◯ Core features: High intake of fruits, vegetables, whole grains, legumes, nuts, and olive oil; moderate consumption of fish, poultry, and dairy; low intake of red meat and sweets; and moderate wine consumption. ◯ Brain health benefits: Rich in antioxidants, polyunsaturated fatty acids (PUFAs), and anti-inflammatory compounds, the MedDiet is associated with reduced risks of cognitive decline, Alzheimer’s disease, and other dementias. DASH diet ◯ Core features: Emphasis on fruits, vegetables, whole grains, low-fat dairy, lean protein, and reduced sodium intake. ◯ Brain health benefits: Known to lower blood pressure, DASH indirectly supports brain health by improving vascular health, which reduces the risk of stroke and vascular dementia. MIND Diet ◯ Core features: Combines elements of the MedDiet and DASH with a focus on specific foods linked to cognitive health, including berries and green leafy vegetables. ◯ Brain health benefits: Developed explicitly for neuroprotection, the MIND diet is associated with slower cognitive decline and lower Alzheimer’s risk, even when adherence is moderate. Western diet ◯ Core features: High intake of processed foods, refined sugars, unhealthy fats, and red/processed meats and low consumption of fruits, vegetables, and whole grains. ◯ Brain health risks: Linked to increased inflammation, oxidative stress, and metabolic syndrome, the Western diet is associated with higher risks of cognitive impairment, dementia, and mental health disorders.
Common Recommendations Across Brain-Healthy Diets High consumption of fruits and vegetables ◯ All three beneficial diets emphasize antioxidant-rich produce to combat oxidative stress. Whole grains and legumes ◯ A shared recommendation to improve vascular health and provide sustained energy. Healthy fats ◯ MedDiet, DASH, and MIND encourage unsaturated fats (olive oil, nuts, seeds) over saturated and trans fats. Reduced red meat ◯ Minimal red and processed meat consumption is a common feature of brain-healthy diets. Low sodium intake ◯ Both DASH and MIND emphasize reduced sodium to lower hypertension risk, indirectly benefiting the brain.
Unique Recommendations MIND diet focuses on specific brain-boosting foods ◯ Includes explicit recommendations for berries and green leafy vegetables, which are not emphasized separately in MedDiet or DASH. Moderate wine consumption ◯ MedDiet includes moderate red wine consumption, which provides resveratrol, though this is not a focus in DASH or MIND. Low-fat dairy in DASH ◯ DASH promotes low-fat dairy, which is not a central component of MedDiet or MIND. Fish and omega-3s ◯ MedDiet prioritizes fish for omega-3 fatty acids more strongly than DASH or MIND.
Interpretation The MedDiet, DASH, and MIND diets converge on recommendations for consuming plant-based foods, healthy fats, and whole grains, while limiting processed foods, unhealthy fats, and red meat. The MIND diet uniquely targets neurodegenerative prevention by emphasizing foods like berries and leafy greens. Conversely, the Western diet’s high intake of processed and inflammatory foods poses significant risks to brain health. Implementing elements from MedDiet, DASH, or MIND can provide a synergistic approach to promoting cognitive health and reducing the risk of dementia.

Abbreviations: MedDiet, the Mediterranean Diet; DASH, Dietary Approaches to Stop Hypertension; MIND, Mediterranean-DASH Intervention for Neurodegenerative Delay.

## Data Availability

No new data were created or analyzed in this study. Data sharing is not applicable to this article.

## Abbreviations

The following abbreviations are used in this manuscript:

ABCDS	Activity, Blood Pressure Control, Connecting, Diet, Sleep
MedDiet	Mediterranean Diet
DASH	Dietary Approaches to Stop Hypertension
MIND	Mediterranean-DASH Intervention for Neurodegenerative Delay

## Appendix A

Full PubMed Search Strategy Organized by PICOS Framework
P—Population
(“Persons”[Mesh] OR “Humans”[Mesh] OR human*[tw] OR people*[tw] OR patient*[tw] OR out-patient*[tw] OR child*[tw] OR adult*[tw] OR aged[tw] OR elderl*[tw] OR age group*[tw] OR individual*[tw] OR adolescent*[tw] OR men[tw] OR women[tw] OR boy[tw] OR boys[tw] OR girl*[tw] OR super-ager*[tw] OR “Aged, 80 and over”[Mesh] OR “Centenarians”[Mesh] OR “Nonagenarians”[Mesh] OR “Octogenarians”[Mesh] OR octogenarian*[tw] OR nonagenarian*[tw] OR semisupercentenarian*[tw] OR semi-supercentenarian*[tw] OR supercentenarian*[tw] OR super-centenarian*[tw] OR oldest old[tw])
I—Intervention/Exposure (Diet)
(“Diet, Healthy”[Mesh] OR healthy diet*[tw] OR healthy eating[tw] OR eating health[tw] OR healthy nutrition[tw] OR prudent diet*[tw] OR “Diet Therapy”[Mesh] OR diet therap*[tw] OR dietary modification*[tw] OR restrictive diet[tw] OR restriction diet therapy[tw] OR restrictive diet therapy[tw] OR dietary restriction*[tw] OR “Dietary Approaches To Stop Hypertension”[Mesh] OR hypertension diet*[tw] OR DASH diet*[tw] OR mediterranean[tw] OR MIND[tw] OR nordic[tw])
C—Comparator (Implicit/General Health Comparisons)
Comparator terms were not explicitly defined in this search due to variability in dietary exposures and health outcomes across studies. Studies with various comparator groups (e.g., low vs. high adherence, different diet types) were included.
O—Outcomes (Brain, Mental, Cognitive, Social Health)
(brain health[tw] OR brain wellbeing[tw] OR brain well-being[tw] OR healthy brain[tw] OR “Healthy Aging”[Mesh] OR healthy aging[tw] OR healthy ageing[tw] OR aging well[tw] OR ageing well[tw] OR well aging[tw] OR well ageing[tw] OR cognitive health[tw] OR healthy cognition[tw] OR mindfulness[tw] OR relaxation[tw] OR peace-of-mind[tw] OR mental health[tw] OR social health[tw] OR mental wellbeing[tw] OR social wellbeing[tw] OR emotional wellbeing[tw] OR mental well-being[tw] OR social well-being[tw] OR emotional well-being[tw] OR mental abuse[tw] OR functional disorder*[tw] OR physical abuse[tw])
OR
(“Dementia”[Mesh] OR dementia*[tw] OR amentia*[tw] OR Alzheimer syndrome[tw] OR Alzheimer-Type Dementia[tw] OR Alzheimer’s disease*[tw] OR Alzheimers disease*[tw] OR Alzheimer dementia*[tw] OR senile dementia[tw] OR senile degenerative dementia[tw] OR Alzheimer sclerosis[tw] OR presenile dementia[tw] OR binswanger disease[tw] OR progressive subcortical encephalopathy[tw] OR binswanger encephalopathy[tw] OR leukoaraiosis[tw] OR subcortical leukoencephalopathy*[tw] OR leukoencephalopathies*[tw] OR binswanger’s encephalopathy[tw] OR binswanger’s disease[tw] OR subcortical vascular dementia[tw] OR progressive aphasia*[tw] OR mesulam’s syndrome[tw] OR “Cognitive Dysfunction”[Majr] OR cognitive dysfunction*[tw] OR cognitive disorder*[tw] OR cognitive impairment*[tw] OR cognitive decline*[tw] OR mental deterioration*[tw])
AND
(“Stroke”[Mesh] OR stroke*[tw] OR cerebrovascular accident*[tw] OR cerebrovascular event*[tw] OR brain attack*[tw] OR cerebrovascular apoplexy[tw] OR brain vascular accident*[tw] OR “CVA”[tw] OR “CVAs”[tw] OR brain infarct*[tw] OR brain venous infarction*[tw] OR venous brain infarction*[tw] OR cerebral circulation infarct*[tw] OR brain circulation infarct*[tw] OR cerebral infarct*[tw] OR choroidal artery infarct*[tw] OR subcortical infarct*[tw] OR multi-infarct dementia*[tw] OR multiinfarct dementia*[tw] OR dementia multi-infarct*[tw] OR lacunar dementia*[tw] OR subarachnoid hemorrhagic[tw] OR brain failure*[tw] OR “Myocardial Ischemia”[Mesh] OR ischemic heart disease*[tw] OR ischaemic heart disease*[tw] OR myocardial ischemia*[tw] OR myocardial ischaemia*[tw] OR coronary heart disease*[tw] OR coronary thrombosis[tw] OR coronary artery disease*[tw] OR coronary atheroscleros*[tw] OR coronary disease*[tw] OR myocardial infarct*[tw] OR heart attack*[tw] OR cardiovascular stroke*[tw] OR “Heart Failure”[Mesh] OR myocardial failure[tw] OR cardiac failure[tw] OR heart decompensation[tw] OR heart failure[tw] OR “Atrial Fibrillation”[Mesh] OR atrial fibrillation*[tw] OR auricular fibrillation*[tw])
S—Study Design
(“Follow-Up Studies”[Mesh] OR follow up stud*[tw] OR followup stud*[tw] OR “Longitudinal Studies”[Mesh] OR longitudinal stud*[tw] OR “Cross-Sectional Studies”[Mesh] OR Cross Sectional Studi*[tw] OR birth cohort*[tw] OR Framingham Heart Stud*[tw] OR Jackson Heart Stud*[tw] OR California Teachers Stud*[tw] OR Bogalusa Heart Stud*[tw] OR Longitudinal Survey*[tw] OR add health study[tw] OR “Prospective Studies”[Mesh] OR “Randomized Controlled Trial”[Publication Type] OR randomized controlled trial*[tw] OR randomised controlled trial*[tw] OR RCT[tw] OR RCTs[tw] OR “Community Health Planning”[mh] OR population based[tw] OR community based[tw] OR community health[tw] OR community dwelling*[tw] OR “Home Care Services, Hospital-Based”[mh] OR hospital based[tw] OR hospital-based[tw] OR clinic based[tw] OR clinic-based[tw]
OR
(meta-analysis[pt] OR meta-analy*[tw] OR metaanaly*[tw] OR meta-synthesis[tw] OR metasynthesis[tw] OR meta-study[tw] OR metastudy[tw] OR metaethnograph*[tw] OR meta-ethnograph*[tw] OR sensitivity analys*[tw] OR systematic review[pt] OR systematic review[tw] OR systematic literature[tw] OR integrative review[tw] OR integrative literature[tw] OR evidence-based review[tw] OR evidence-based overview[tw] OR evidence-based literature[tw] OR evidence-based survey[tw] OR literature search[tw])
OR
((systemat*[ti] OR evidence-based[ti]) AND (review*[ti] OR literature[ti] OR overview[ti] OR survey[ti]))
OR
data synthesis[tw] OR evidence synthesis[tw] OR data extraction[tw] OR data source[tw] OR data sources[tw] OR study selection[tw] OR methodological quality[tw] OR methodologic quality[tw] OR cochrane database syst rev[ta] OR umbrella review[pt] OR “Comparative Effectiveness Research”[mh]
OR
(“Life Change Events”[Mesh] OR life change event*[tw] OR life crisis[tw] OR life course*[tw] OR life experience*[tw]))
